# Case Report: Focal, generalized, or both: does generalized network involvement preclude successful epilepsy surgery?

**DOI:** 10.3389/fnetp.2024.1425329

**Published:** 2024-07-11

**Authors:** Cathy K. Cui, Wui-Kwan Wong, Chong H. Wong, Deepak Gill, Michael W. K. Fong

**Affiliations:** ^1^ Westmead Comprehensive Epilepsy Centre, The University of Sydney, Sydney, NSW, Australia; ^2^ T. Y. Nelson Department of Neurology and Neurosurgery, The Children’s Hospital at Westmead, Sydney, NSW, Australia; ^3^ Department of Neurology, Comprehensive Epilepsy Center, Yale University School of Medicine, New Haven, CT, United States

**Keywords:** epileptic networks, epilepsy surgery, intracranial electroencephalography, frontal lobe epilepsy, semiology

## Abstract

We present two cases with focal seizures where scalp electroencephalography (EEG) had prominent features of a developmental and epileptic encephalopathy (DEE): Case 1: a 17-year-old male with complex motor seizures whose EEG demonstrated a slow spike-and-wave pattern and generalized paroxysmal fast activity (GPFA). Case 2: a 12-year-old male with startle-induced asymmetric tonic seizures whose EEG also had a slow spike-and-wave pattern. Both patients had intracranial EEG assessment, and focal cortical resections resulted in long-term seizure freedom and resolution of generalized findings. These cases exemplify patients with focal epilepsy with networks that share similarities to generalized epilepsies, and importantly, these features did not preclude curative epilepsy surgery.

## 1 Introduction

Two decades ago, Spencer published seminal works proposing the “network theory” of epilepsy (Spencer, 2002). The network theory put forward that focal epilepsies are not so focal but rather result from the activation of broader networks of varying scales (Spencer, 2002). This theory expanded the historical Lüders’ definition of the epileptogenic zone as “the minimum amount of the cortex that must be resected to produce seizure freedom” (Luders et al., 2006). Spencer et al. argued that “the network as a whole is responsible for the clinical and electrographic phenomena that we associate with human seizures” (Spencer, 2002). This shift in concept paved the way for now routine components of the pre-surgical evaluation for patients with drug-resistant epilepsy (DRE), including the utilization of functional imaging to further define epilepsy networks. With this more global view in mind, certain nodes or connections may still be more epileptogenic than others. Bartolomei et al. (2017) defined this hierarchical organization of focal epilepsy in their model, differentiating between “epileptogenic zone networks,” “propagation zone networks,” and non-involved networks. It has since been established that how distributed an epilepsy network is for a given patient is a determinant of successful epilepsy surgery, with more restricted networks that are slow to recruit distant regions doing better than those that rapidly recruit distant or bilateral nodes (Andrews et al., 2019).

A rare phenomenon is the early recruitment of distant and bilateral structures by a focal epilepsy, giving the electroclinical appearance of an apparent generalized epilepsy syndrome. This paper highlights two such cases and discusses the conceptual mechanism underlying such findings while exploring factors that prompted intracranial EEG assessment and successful epilepsy surgery.

## 2 Case studies

### 2.1 Case 1

Case 1 was a 17-year-old, left-handed male patient with normal development who was diagnosed with epilepsy at the age of 13 years. He presented with focal seizures with impaired awareness (FIA), with a preceding aura of a warm sensation in his back which might cause him to perspire. This was followed by loss of responsiveness and rapid, repeated semi-purposeful upper limb movements, such as clapping, tapping, leg-slapping, scratching, and grabbing. There was a change in facial expression with an asymmetric grimace, and the head and eyes would turn in a non-forced fashion to the right. The left upper limb would occasionally posture, but this was not clearly dystonic, and at no time would he develop clonic jerking of the arm. Each discrete seizure was short (often 5–15 s); however, he often developed seizure clusters where he could have many over a 20-min period. He was immediately responsive in between seizures but experienced tiredness after seizure clusters. He had developed a second seizure type 3 years into his condition, with frequent brief tonic seizures during sleep. He did not have a history of febrile seizures during childhood or a family history of epilepsy. His neurological examination was normal. Prolonged seizure clusters occurred several times a month despite combination treatment with carbamazepine, lamotrigine, and clobazam. He had previously failed multiple medication trials including sodium valproate, levetiracetam, and topiramate.

**FIGURE 1:**
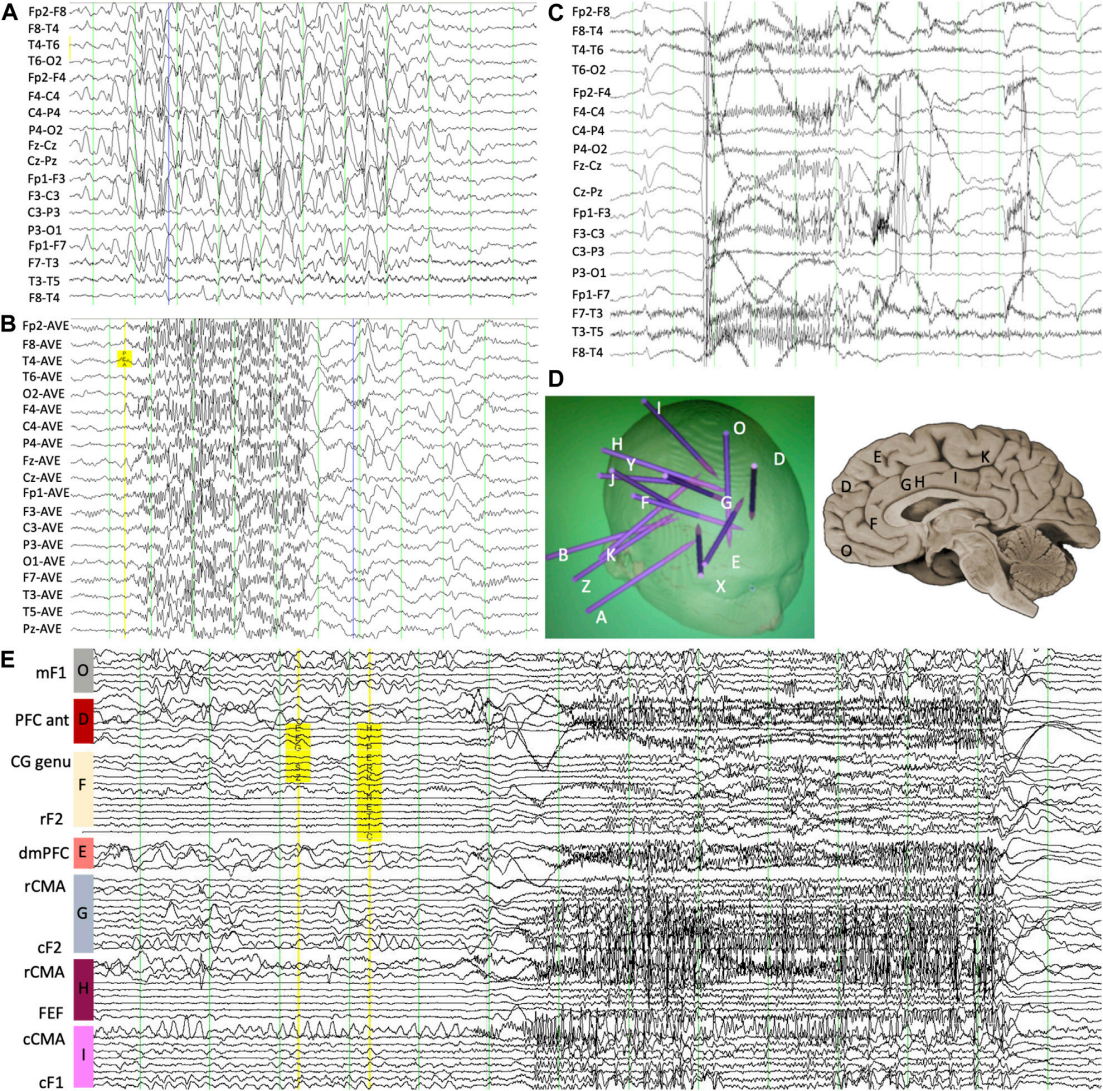
**(A)** Scalp EEG demonstrating interictal slow spike-and-wave with an internal frequency of 1.5 Hz and **(B)** GPFA though with increased amplitude over the right frontal region. **(C)** Ictal scalp EEG showing evolving low-amplitude fast activity at the onset of the complex motor seizure. **(D)** Intracranial depth electrode monitoring placement map showing overall implantation strategy as well as locations of mesial-frontal contacts. **(E)** Ictal stereo-EEG confirming a focal onset at the right cingulate gyrus with the patient’s typical seizure, occurring 1 s before broad network involvement. mF1, mesial superior frontal gyrus; PFC ant, anterior prefrontal cortex; CG genu, pregenual cingulate gyrus; rF2, rostral middle frontal gyrus; dmPFC, dorsomedial prefrontal cortex; rCMA, rostral cingulate motor area; cF2, caudal middle frontal gyrus; FEF, frontal eye field; cCMA, caudal cingulate motor area; cF1, caudal superior frontal gyrus.

Video electroencephalography (VEEG) demonstrated frequent interictal generalized paroxysmal fast activity (GPFA) and generalized slow spike/poly-spike-and-wave discharges but with a consistent right fronto-central predominance (Figure 1). Multiple seizures were recorded, during which the rapid hyperkinetic movements correlated with 20–24 Hz GPFA, similar to the expected EEG pattern of tonic seizures in patients with Lennox–Gastaut syndrome (LGS) (Markand, 2003). Brain magnetic resonance imaging (MRI) and fludeoxyglucose (FDG)-PET were normal. Despite all the scalp EEG findings being suggestive of generalized epilepsy with normal imaging, there was a high suspicion for a focal epilepsy syndrome given the consistent aura. He proceeded to an intracranial EEG implantation via stereo-EEG (SEEG) at age 16 years to explore the right frontal region and particularly the mesial frontal region, cingulate, and insula. Both ictal recordings and intracranial stimulation confirmed that his seizures were focal and arose from the right cingulate gyrus (Figure 1), characterized by low-voltage fast activity first developing in the right cingulate contacts before spread to the remainder of the frontal networks occurring between 1 and 2 seconds into the seizure. He proceeded to right mesial frontal surgical resection, which showed focal cortical dysplasia (FCD) type 1a. He has remained seizure-free at 8 years of follow-up (Engel-Ia), although he remains on reduced anti-seizure medication (ASM). His EEG at 6 months after surgery demonstrated marked improvement with only occasional right fronto-temporal sharp waves with some associated fast activity but complete resolution of the GPFA and generalized slow spike-and-wave.

### 2.2 Case 2

Case 2 was a 12-year-old, right-handed male patient with a history of reflex epilepsy from age 6 years. He had mild delay in gross motor and social milestones prior to the onset of seizures but had clear regression in behavior and learning following the development of epilepsy. There was no history of perinatal insult or febrile convulsion. Although his seizures were predominantly nocturnal, when awake, they would be triggered by loud noises, surprise, or fright. FIA would start with a grimace or odd facial expression, followed by asymmetric stiffening of the trunk and limbs (particularly the right leg) and truncal rocking lasting 15–45 s. He occasionally reported an aura of “fireworks” or a “booming” body sensation prior to a seizure. Seizures remained at least once every 2 weeks on a combination of topiramate, carbamazepine, and clobazam.

**FIGURE 2:**
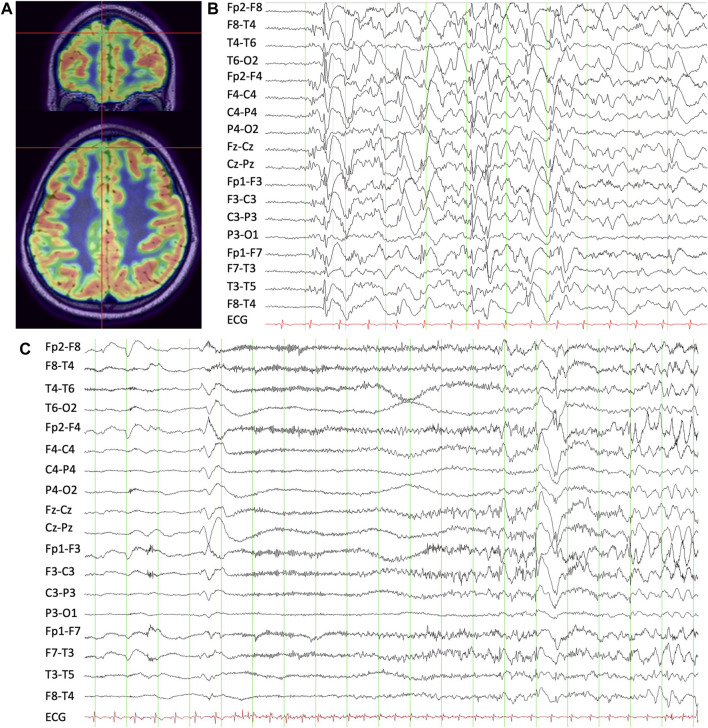
**(A)** T1 MRI-brain overlaid with FDG-PET demonstrating right mesial superior frontal gyrus hypometabolism. **(B)** Interictal scalp EEG showing apparent generalized slow spike-and-wave discharges with a right frontal predominance. **(C)** Ictal scalp EEG during a habitual asymmetric tonic seizure showing evolving low-amplitude fast activity with aftercoming slowing.

Like Case 1, VEEG demonstrated generalized slow spike-and-wave discharges with a right frontal predominance (Figure 2). The scalp EEG correlate to the seizures described above was also electro-decrement with evolving low-voltage fast activity for 10–15 s followed by aftercoming irregular delta slowing. MRI brain was non-lesional. FDG-PET demonstrated mild hypometabolism of the right mesial superior frontal gyrus (Figure 2). Again, given the focal seizure semiology with an aura, as well as consistently lateralized right leg dystonic posturing, he proceeded to SEEG of the right frontal region, also including cingulate and insula. Several habitual asymmetric tonic seizures were recorded. The SEEG onset was broad, but there was a consistent lead with fast activity over-riding direct current (DC) shift at several contacts in the mesial prefrontal region. High-frequency oscillations were present in that region and also in the orbitofrontal gyrus. Seizure stimulation across the posterior dorsolateral superior frontal gyrus and pre-supplementary motor area (pre-SMA) reproduced his typical electroclinical seizures. He proceeded to a mesial prefrontal resection of the superior frontal gyrus up to the precentral sulcus, which showed neuronal loss and gliosis in keeping with distant infarction. He has remained seizure-free at 10-year follow-up (Engel Ia), and his EEG repeated at 8 months following surgery was normal.

## 3 Discussion

These two cases highlight patients with focal epilepsy presenting with EEG features of generalized epilepsy syndromes. Both patients developed clinical features that are more common in generalized epilepsy, such as tonic seizures in Case 1 or a prominent reflex component in Case 2. The MRI of both patients was non-lesional. Both patients had mesial frontal epilepsy confirmed with SEEG (Case 1: cingulate; Case 2: pre-SMA). This has potential relevance to the likelihood of networks with shared similarities to those with generalized epilepsy; however, such recruitment did not seem to preclude the possibility of successful epilepsy surgery.

There is a great overlap between the seizure networks described in frontal lobe epilepsy and that of generalized epilepsy. In frontal lobe epilepsy, typically complex motor or hyperkinetic seizures are associated with the engagement of wide-spread networks through both hemispheres (Chauvel and McGonigal, 2014), compared to seizures with elementary or even autonomic signs having highly specific cortical localization that can be reliably reproduced with cortical stimulation (McGonigal et al., 2021). Case 1 had seizures arising from the cingulate gyrus, with rapid goal-directed behaviors subsequently described as being typical of the anterior mid-cingulate region (Pelliccia et al., 2022), and Case 2 had pre-SMA onsets. In each case, there was broad activation through frontal networks, and these recruitments often occurred between 1 and 2 seconds following seizure onset.

These findings can be related to the current understanding of the traditional generalized epilepsies. For patients with idiopathic generalized epilepsy (IGE), EEG-functional MRI (EEG-fMRI) studies have implicated bilateral cortico-subcortical networks, consisting of mid-frontal regions, thalami, caudate, and cerebellum (Aghakhani et al., 2004; Hamandi et al., 2008). When assessed with magnetoencephalography (MEG), generalized epileptiform discharges were derived from bilateral mesial-frontal regions (Li et al., 2018). For patients with DEEs, particularly those with LGS, an EEG-fMRI study showed that the network activation was different for the generation of slow spike-and-wave versus GPFA (Pillay et al., 2013). Both involved diffuse bilateral networks; however, the activations during GPFA were almost exclusively positive, affecting broad cortical “association” areas in frontal, parietal, occipital, and temporal lobes but not the primary cortices (Pillay et al., 2013). In addition, there were synchronous increases in the thalamus, caudate-basal ganglia, and brainstem (Pillay et al., 2013). Conversely, slow spike-and-wave generated a mixture of positive and negative blood oxygen level-dependent (BOLD) signal changes in the brain regions discussed and was overall much more variable between patients (Pillay et al., 2013).

Overall, the consistent feature with these studies was that the generalized epilepsy network had broad involvement of prefrontal and mesial-frontal regions (excluding primary motor cortex), when—typically for focal networks—seizure activity remains confined to a smaller cluster or subnetwork of the larger global brain network (Woldman et al., 2020). However, there is clearly a dynamic component, given that the same structures operate interictally during normal function (Richardson, 2012). In addition, the pathophysiology of widespread synchronization is still elusive. Instead of being “non-focal,” the apparent synchronous character of generalized discharges in a genetic rat model of absence seizures arises from extremely fast cortical spread of seizure activity from a localized cortical focus, which also drives initial rhythmicity (Meeren et al., 2002). Focal lesions may also persistently engage with these networks over time, leading to global network instability rather than driving each individual discharge (Archer et al., 2014). Given the network overlaps, frontal lobe epilepsy tends to involve existing widespread brain networks (Bancaud et al., 1974; Kakisaka et al., 2011). This contrasts with temporal lobe epilepsy with few examples of generalized network recruitment in the literature; if generalized EEG changes are present, they often occur in the context of co-existent focal and generalized epilepsy (Khaing et al., 2014). Though there are no clear predictors for such network involvement, lesions arising in anatomical brain regions that are already richly bilaterally connected (e.g., frontal and cingulate regions) appear to be more vulnerable (Tukel and Jasper, 1952; Blumenfeld et al., 2009; Chen et al., 2016). Abnormal connectivity also changes over time and is influenced by other factors such as age, duration of epilepsy, and seizure frequency (Javidi et al., 2024).

There are two highly relevant clinical pearls when it comes to these cases: 1. patients with focal lesions can develop clinical manifestations of generalized epilepsy with recruitment of other structures in an individual’s broader epilepsy network; 2. a patient’s overall pathologic network activation can be disrupted if a crucial driving “node” in that network is removed.

Case 1 subsequently developed tonic seizures. Tonic seizures with an ictal pattern of GPFA is one of the electroclinical hallmarks of LGS but can develop in patients with focal epilepsies, especially those without the pre-requisite developmental encephalopathy (Dupont et al., 2017). In focal cases, the PFA tended to have a bi-frontal predominance (regardless of the location of the lesion), but it was asymmetrical with higher voltage over the implicated hemisphere (Mohammadi et al., 2015). Our two cases are particularly striking. Both patients had clearly focal seizures, but the electrographic correlate to these seizures was evolving low-amplitude fast activity with aftercoming slowing (the EEG signature of tonic seizures). Case 2 had a prominent reflex component and regression in learning and behavior after the development of epilepsy. Startle-induced seizures with bilateral asymmetric tonic features are thought to involve several brain regions in the mesial-frontal and parietal lobes, with a common final pathway to the supplementary motor area (Job et al., 2014). Functional neuroimaging suggests that these could be generated by a frontal–parietal network located over the mesial surface of the brain, involving the precuneus, supplementary motor area, cingulate gyrus, and precentral regions (Fernandez et al., 2011). The theme continues: the brain regions involved in our patients had a great overlap with the generalized network and those implicated in startle-induced seizures (a clinical feature more common in the DEEs).

In the pre-surgical assessment for epilepsy surgery, clinical features usually attributed to generalized epilepsy such as tonic seizures (to a lesser extent reflex or startle-induced seizures) or EEG features such as GPFA and slow spike-and-wave have often precluded further invasive workup. It is true that in patients with focal epilepsy, the presence of generalized EEG features negatively impacts the likelihood of successful epilepsy surgery (Janszky et al., 2000); however, as shown by our two patients, these features do not guarantee failure at all.

In both cases, the seizure semiology showed focal features, which provided the greatest support for a focal “node” in a broader epilepsy network (even though all other non-invasive pre-surgical data were generalized). Both patients had diffuse pathologies upon resection, namely, FCD type 1a in Case 1 and gliosis suggestive of old infarction in Case 2. Resection in both cases resulted in prolonged seizure freedom (Engel Ia). Importantly, this also meant free of all seizures including those that subsequently developed (i.e., tonic seizures in Case 1). In addition to this, all of the EEG features of generalized epilepsy completely resolved following epilepsy surgery, proving the greatest evidence that normalization can occur if the focal driving force in a patient’s broader epilepsy network is subsequently removed (Archer et al., 2014). Findings from our cases are not, however, generalizable to most focal epilepsies: in fact, diffuse pathologies such as FCD type 1 tend to be associated with both secondary bilateral synchrony and surgical failure (Krsek et al., 2008), with additional factors such as older age at epilepsy surgery and multi-lobar extent of pathology modulating likelihood of seizure freedom (Fauser et al., 2008).

## 4 Conclusion

These two cases highlight that focal epilepsy with broad bilateral networks even representing those seen in generalized epilepsy is still amenable to curative epilepsy surgery. Each case was assessed with stereo-EEG, and focal resections resulted in complete seizure freedom (of all types of seizures) and containment of bilateral network activation. They form the framework to discuss generalized network recruitment and emphasize that with careful patient selection, good clinical outcomes are still possible.

## References

[B2] Aghakhani Y. Bagshaw A. P. Benar C. G. Hawco C. Andermann F. Dubeau F. (2004). fMRI activation during spike and wave discharges in idiopathic generalized epilepsy. Brain 127 (Pt 5), 1127–1144. 10.1093/brain/awh136 15033899

[B3] Andrews J. P. Gummadavelli A. Farooque P. Bonito J. Arencibia C. Blumenfeld H. (2019). Association of seizure spread with surgical failure in epilepsy. JAMA Neurol. 76 (4), 462–469. 10.1001/jamaneurol.2018.4316 30508033 PMC6459131

[B4] Archer J. S. Warren A. E. Jackson G. D. Abbott D. F. (2014). Conceptualizing lennox-gastaut syndrome as a secondary network epilepsy. Front. Neurol. 5, 225. 10.3389/fneur.2014.00225 25400619 PMC4214194

[B5] Bartolomei F. Lagarde S. Wendling F. McGonigal A. Jirsa V. Guye M. (2017). Defining epileptogenic networks: contribution of SEEG and signal analysis. Epilepsia 58 (7), 1131–1147. 10.1111/epi.13791 28543030

[B1] Bancaud J. Talairach J. Morel P. Bresson M. Bonis A. Geier S. (1974). “Generalized” epileptic seizures elicited by electrical stimulation of the frontal lobe in man. Electroencephalogr. Clin. Neurophysiol. 37 (3), 275–282. 10.1016/0013-4694(74)90031-5 4136279

[B6] Blumenfeld H. Varghese G. I. Purcaro M. J. Motelow J. E. Enev M. McNally K. A. (2009). Cortical and subcortical networks in human secondarily generalized tonic-clonic seizures. Brain 132 (Pt 4), 999–1012. 10.1093/brain/awp028 19339252 PMC2724910

[B7] Chauvel P. McGonigal A. (2014). Emergence of semiology in epileptic seizures. Epilepsy Behav. 38, 94–103. 10.1016/j.yebeh.2013.12.003 24424286

[B8] Chen P. C. Castillo E. M. Baumgartner J. Seo J. H. Korostenskaja M. Lee K. H. (2016). Identification of focal epileptogenic networks in generalized epilepsy using brain functional connectivity analysis of bilateral intracranial EEG signals. Brain Topogr. 29 (5), 728–737. 10.1007/s10548-016-0493-3 27142358

[B9] Dupont S. Banica-Wolters R. An-Gourfinkel I. Lambrecq V. Navarro V. Adam C. (2017). Understanding Lennox-Gastaut syndrome: insights from focal epilepsy patients with Lennox-Gastaut features. J. Neurol. 264 (7), 1388–1396. 10.1007/s00415-017-8535-7 28584915

[B10] Fauser S. Bast T. Altenmuller D. M. Schulte-Monting J. Strobl K. Steinhoff B. J. (2008). Factors influencing surgical outcome in patients with focal cortical dysplasia. J. Neurol. Neurosurg. Psychiatry 79 (1), 103–105. 10.1136/jnnp.2007.116038 17682011

[B11] Fernandez S. Donaire A. Maestro I. Seres E. Setoain X. Bargallo N. (2011). Functional neuroimaging in startle epilepsy: involvement of a mesial frontoparietal network. Epilepsia 52 (9), 1725–1732. 10.1111/j.1528-1167.2011.03172.x 21770921

[B12] Hamandi K. Laufs H. Noth U. Carmichael D. W. Duncan J. S. Lemieux L. (2008). BOLD and perfusion changes during epileptic generalised spike wave activity. Neuroimage 39 (2), 608–618. 10.1016/j.neuroimage.2007.07.009 17920297

[B13] Janszky J. Jokeit H. Schulz R. Hoppe M. Ebner A. (2000). EEG predicts surgical outcome in lesional frontal lobe epilepsy. Neurology 54 (7), 1470–1476. 10.1212/wnl.54.7.1470 10751260

[B14] Javidi S. S. He X. Ankeeta A. Zhang Q. Citro S. Sperling M. R. (2024). Edge-wise analysis reveals white matter connectivity associated with focal to bilateral tonic-clonic seizures. Epilepsia 65, 1756–1767. 10.1111/epi.17960 38517477 PMC11166520

[B15] Job A. S. De Palma L. Principe A. Hoffmann D. Minotti L. Chabardes S. (2014). The pivotal role of the supplementary motor area in startle epilepsy as demonstrated by SEEG epileptogenicity maps. Epilepsia 55 (8), e85–e88. 10.1111/epi.12659 24902865

[B16] Kakisaka Y. Alexopoulos A. V. Gupta A. Wang Z. I. Mosher J. C. Iwasaki M. (2011). Generalized 3-Hz spike-and-wave complexes emanating from focal epileptic activity in pediatric patients. Epilepsy Behav. 20 (1), 103–106. 10.1016/j.yebeh.2010.10.025 21131239 PMC3992252

[B17] Khaing M. Lim K. S. Tan C. T. (2014). Focal epilepsy recruiting a generalised network of juvenile myoclonic epilepsy: a case report. Epileptic Disord. 16 (3), 370–374. 10.1684/epd.2014.0672 25166001

[B18] Krsek P. Maton B. Korman B. Pacheco-Jacome E. Jayakar P. Dunoyer C. (2008). Different features of histopathological subtypes of pediatric focal cortical dysplasia. Ann. Neurol. 63 (6), 758–769. 10.1002/ana.21398 18571798

[B19] Li H. Y. Marquetand J. Elshahabi A. Klamer S. Lerche H. Braun C. (2018). Increased functional MEG connectivity as a hallmark of MRI-negative focal and generalized epilepsy. Brain Topogr. 31 (5), 863–874. 10.1007/s10548-018-0649-4 29766384

[B20] Luders H. O. Najm I. Nair D. Widdess-Walsh P. Bingman W. (2006). The epileptogenic zone: general principles. Epileptic Disord. 8 (Suppl. 2), S1–S9. 10.1684/j.1950-6945.2006.tb00204.x 17012067

[B21] Markand O. N. (2003). Lennox-Gastaut syndrome (childhood epileptic encephalopathy). J. Clin. Neurophysiol. 20 (6), 426–441. 10.1097/00004691-200311000-00005 14734932

[B22] McGonigal A. Bartolomei F. Chauvel P. (2021). On seizure semiology. Epilepsia 62 (9), 2019–2035. 10.1111/epi.16994 34247399

[B23] Meeren H. K. Pijn J. P. Van Luijtelaar E. L. Coenen A. M. Lopes da Silva F. H. (2002). Cortical focus drives widespread corticothalamic networks during spontaneous absence seizures in rats. J. Neurosci. 22 (4), 1480–1495. 10.1523/JNEUROSCI.22-04-01480.2002 11850474 PMC6757554

[B24] Mohammadi M. Okanishi T. Okanari K. Baba S. Sumiyoshi H. Sakuma S. (2015). Asymmetrical generalized paroxysmal fast activities in children with intractable localization-related epilepsy. Brain Dev. 37 (1), 59–65. 10.1016/j.braindev.2014.03.006 24809226

[B25] Pelliccia V. Avanzini P. Rizzi M. Caruana F. Tassi L. Francione S. (2022). Association between semiology and anatomo-functional localization in patients with cingulate epilepsy: a cohort study. Neurology 98 (22), e2211–e2223. 10.1212/WNL.0000000000200145 35190463

[B26] Pillay N. Archer J. S. Badawy R. A. Flanagan D. F. Berkovic S. F. Jackson G. (2013). Networks underlying paroxysmal fast activity and slow spike and wave in Lennox-Gastaut syndrome. Neurology 81 (7), 665–673. 10.1212/WNL.0b013e3182a08f6a 23864316 PMC3775693

[B27] Richardson M. P. (2012). Large scale brain models of epilepsy: dynamics meets connectomics. J. Neurol. Neurosurg. Psychiatry 83 (12), 1238–1248. 10.1136/jnnp-2011-301944 22917671

[B28] Spencer S. S. (2002). Neural networks in human epilepsy: evidence of and implications for treatment. Epilepsia 43 (3), 219–227. 10.1046/j.1528-1157.2002.26901.x 11906505

[B29] Tukel K. Jasper H. (1952). The electroencephalogram in parasagittal lesions. Electroencephalogr. Clin. Neurophysiol. 4 (4), 481–494. 10.1016/0013-4694(52)90079-5 12998596

[B30] Woldman W. Schmidt H. Abela E. Chowdhury F. A. Pawley A. D. Jewell S. (2020). Dynamic network properties of the interictal brain determine whether seizures appear focal or generalised. Sci. Rep. 10 (1), 7043. 10.1038/s41598-020-63430-9 32341399 PMC7184577

